# Silylated-Acetylated Cyclodextrins as Chiral Sensors for the Enantiodiscrimination of Fluorinated Anesthetics

**DOI:** 10.3390/molecules28062804

**Published:** 2023-03-20

**Authors:** Alessandra Recchimurzo, Federica Balzano, Gloria Uccello Barretta, Luca Gherardi, Milo Malanga, Federica Aiello

**Affiliations:** 1 Department of Chemistry and Industrial Chemistry, University of Pisa, via G. Moruzzi 13, 56124 Pisa, Italy; 2 CycloLab, Cyclodextrin R&D Ltd., Illatos út. 7, H-1097 Budapest, Hungary; 3 National Research Council, Institute for Chemical and Physical Processes (CNR-IPCF), via G. Moruzzi 1, 56124 Pisa, Italy

**Keywords:** NMR, chiral solvating agents, ROESY, isoflurane, desflurane, halothane, enflurane, supramolecular interactions

## Abstract

Silylated-acetylated cyclodextrin (CD) derivatives have recently been investigated, via nuclear magnetic resonance (NMR) spectroscopy, as chiral sensors for substrates that are endowed and devoid of fluorine atoms, and the importance of Si-F interaction in the discrimination phenomena has been assessed. Here, the contributions of both superficial interactions and inclusion processes were further evaluated by extending the records to other chiral fluorinated substrates of interest for pharmaceutical applications. Non-equivalences were measured for both the ^1^H and ^19^F resonances in equimolar mixtures with the CDs; the promising results also supported the use of chiral sensors in *sub*-stoichiometric amounts. Finally, the occurrence of inclusion processes was evaluated by analyzing the intermolecular dipolar interactions by means of ROESY (Rotating-frame Overhauser Enhancement Spectroscopy) experiments. The study confirmed that the γCD derivative is the best chiral solvating agent for the fluorinated substrates investigated, likely due to the higher number of silyl moieties that can be involved in Si-F interactions. The contribution of inclusion processes to the enantiodiscrimination was also confirmed by comparison with the α- and β-analogues. Overall, the CD derivatives proved to be able to discriminate fluorinated substrates even when used in *sub*-stoichiometric amounts.

## 1. Introduction

Cyclodextrins (CDs) are chiral cyclic oligosaccharides consisting of six, seven or eight units of d-glucopyranose linked via α-1,4 glycosidic bonds [1]. Thanks to their supramolecular arrangement, they are widely applied as solubility and stability enhancers in aqueous media. Indeed, the presence of three hydroxyl functionalities in native CDs favors the formation of hydrogen-bond networks that reflect into a truncated-cone shape structure and are endowed with a hydrophobic cavity in which molecules with scarce aqueous solubility can be accommodated. The three hydroxyl groups of the glucopyranose unit have different reactivity, and their functionalization can lead to a wide range of CD derivatives with different solubility features and complexing properties [1,2,3]. Depending on the nature of the derivatizing groups, CD derivatives can be employed in both polar and apolar media.

Thanks to their chirality, derivatized and native cyclodextrins can give rise to stereoselective recognition phenomena, which can be exploited in asymmetric syntheses [4,5] in order to design chromatographic separation systems [6,7,8,9,10] or as chiral solvating agents (CSAs) for nuclear magnetic resonance (NMR) spectroscopy [4,6,7,8,9,10,11,12,13].

**Figure 1:**
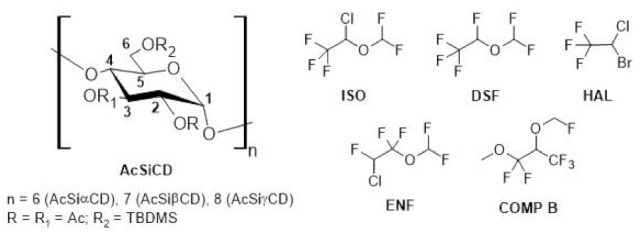
Chemical structures of acetylated-silylated cyclodextrin derivatives and fluorinated compounds selected for the NMR study. Ac and TBDMS stand for acetyl and *tert*-butyldimethylsilyl, respectively.

Among different cyclodextrin derivatives, lipophilic ones have led to the design of promising analytical and preparative chromatographic separation systems in apolar media based on chemo- and stereoselective molecular recognition [14,15,16,17,18,19]. In addition, among lipophilic derivatives, (2,3-di-*O*-acetyl-6-*O*-*tert*-butyldimethylsilyl)cyclodextrins (AcSiCDs, Figure 1) have been tested as CSAs for NMR spectroscopy [20,21], and, more recently, comparisons have been made of the enantiodifferentiation of substrates that are endowed and devoid of fluorine atoms, specifically 1,1,1,3,3-pentafluoro-2-(fluoromethoxy)-3-methoxypropane (COMP B in Figure 1) and methyl 2-chloropropionate [22]. The authors of the NMR study [22] pointed out a competition between a deep inclusion within the host’s cavity, the superficial interactions involving the fluorine atoms of COMP B and the *tert*-butyldimethylsilyl derivatizing groups on the primary side of the CDs. The γ-derivative was able to generate the most efficient differentiation between the two enantiomers of COMP B.

In the light of results described above, the use of the three AcSiCDs as CSAs was extended to other pharmacologically relevant chiral fluorinated anesthetics, namely isoflurane (ISO), desflurane (DSF), halothane (HAL) and enflurane (ENF) (Figure 1). Firstly, equimolar CSA/substrate conditions were evaluated, and, subsequently, *sub*-stoichiometric amounts of the CSAs were considered. Indeed, the latter approach is very attractive for chiral auxiliaries characterized by high levels of symmetry and medium/high molecular weights, as in the case of cyclodextrin macrocycles, since these translate into lower CSA consumption and into a reduction of spectral interference from CSA signals in the NMR spectra [23,24,25]. For the sake of comparison, *sub*-stoichiometric conditions were also considered in the case of COMP B, which was previously analyzed in an equimolar mixture by exploiting the NMR of the proton [22].

The enantiodiscrimination phenomena were investigated by measuring the non-equivalence (|δ*_R_* − δ*_S_*|, the difference in ppm observed between the chemical shifts of the signals due to the two enantiomers of the substrates in the presence of the CSA) experienced by both the proton (^1^H) and the fluorine (^19^F) nuclei of the substrates in host/guest mixtures prepared at different molar ratios. The origin of chiral discrimination processes was investigated thoroughly from a stereochemical point of view by detecting the intermolecular dipolar interactions via ROESY (Rotating-frame Overhauser Enhancement SpectroscopY) experiments [26,27]. Deuterated cyclohexane (C_6_D_12_), which had already been exploited as a solvent in the analysis of chiral discrimination phenomena generated by AcSiCDs [22], was selected for this NMR study, considering that it could potentially favor the host/guest interactions and that it effectively mimics the apolar gas chromatographic environment [14,15,16,17,18,19].

## 2. Results and Discussion

Enantiodiscrimination processes were evaluated in mixtures containing fixed amounts of each fluorinated anesthetic and variable amounts of the host in C_6_D_12_.

### 2.1. NMR Enantiodiscrimination Experiments of Isoflurane (ISO)

Preliminarily, 30 mM equimolar solutions of isoflurane/AcSiCDs were analyzed (Table 1), and then the CD concentration was reduced to reach an equimolar ratio between the substrate and one glucopyranose unit; therefore, a 6-to-1 ISO/CD ratio was used in the case of the α-derivative, which translates into a cyclodextrin concentration of 5 mM. Analogously, the AcSiβCD concentration was lowered to 4.3 mM, corresponding to a 7-to-1 ISO/CD ratio, and the AcSiγCD was altered to 3.75 mM (8-to-1 ISO/CD ratio). In addition, 3-to-1, 3.5-to-1 and 4-to-1 ISO/CD mixtures were analyzed for the α-, β- and γ-derivative, respectively; these molar ratios corresponded to the presence of two equivalents of glucosidic units for one equivalent of substrate.

The highest non-equivalences for ISO resonances were achieved in the presence of the largest macrocycle (Table 1), as already observed with COMP B [22]. The enantiomeric differentiation of protons was remarkably lower in the presence of one equivalent of AcSiβCD, and no effect was observed in the corresponding mixture with AcSiαCD. Regarding the fluorine nuclei, the CHF_2_ group was affected by the interactions with every CD derivative, even though it was to a lower extent for the α- and β-analogues than the γ-derivative (Table 1).

**Figure 2:**
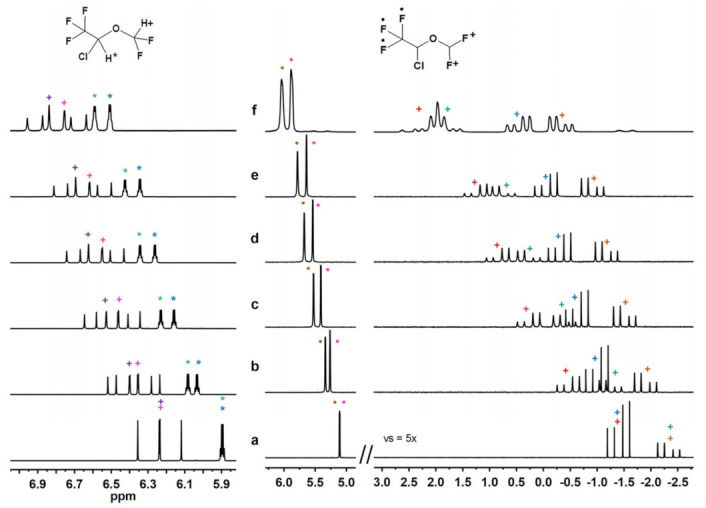
^1^H (600 MHz, 25 °C, C_6_D_12_) (**left**) and ^19^F (564 MHz, 25 °C, C_6_D_12_) (**right**) NMR spectra of racemic ISO (30 mM) alone (**a**) and in the presence of AcSiγCD to give an ISO/CD molar ratio of 8:1 (**b**), 4:1 (**c**), 3:1 (**d**), 2:1 (**e**) and 1:1 (**f**). The different colors help in visualizing the splitting of the signals as a consequence of the interaction with the host.

Focusing on the 1-to-1 ISO/AcSiγCD mixture (Table 1 and Figure 2), similarly high non-equivalences were measured for the two methine protons of the substrate (0.083 ppm and 0.082 ppm), whereas strongly differentiated non-equivalences were detected in the two different kinds of fluorinated probes (CF_3_ and CHF_2_). A non-equivalence of 0.165 ppm was measured for the CF_3_ nuclei, and remarkable differentiations of 1.98 ppm and 2.08 ppm were observed in the two diastereotopic fluorines belonging to the CHF_2_ group (Table 1).

Interestingly, lowering the amount of CD had negligible effect on the non-equivalences up to an ISO/CD ratio corresponding to 4-to-1 (namely, two glucopyranose units per equivalent of ISO), where the differentiations of the CHCF_3_ and CHF_2_ protons underwent reductions of only about 14% and 21%, respectively. Overall, proton non-equivalences were still appreciable in *sub*-stoichiometric conditions up to the 8-to-1 ISO/CD ratio (Table 1 and Figure 2). It is worth mentioning that high non-equivalences were detected also in the presence of 1/16 equivalents of γ-cyclodextrin with CHCF_3_ and CHF_2_ proton values of 0.030 ppm and 0.027 ppm, respectively (Figure S1, Supplementary Materials).

With regards to the ^19^F nuclei, the enantiomeric differentiation diminished by about 30% for the CF_3_ group at a 4-to-1 ratio, while reductions of about 46% and 55% were observed for the two diastereotopic fluorines, for which the non-equivalences detected were still remarkably high (1.12 ppm and 0.894 ppm, Table 1 and Figure 2).

As expected, the reduction of the total concentration to 10 mM affected the enantiodiscrimination, causing lower non-equivalences; the extent of these decreases depended on the size of the macrocycle. As a matter of fact, an average drop of 30% in non-equivalences was observed in case of AcSiγCD, whereas a 50% reduction was measured in ISO non-equivalences in the equimolar mixture with AcSiβCD (Table S1, Supplementary Material). This trend confirmed that interactions with the γ-CD derivative are stronger and that the chiral discrimination is still relevant in spite of a one-third concentration reduction.

### 2.2. NMR Enantiodiscrimination Experiments with the Other Substrates

By using the same analytical approach described for ISO, the fluorinated compounds DSF, HAL and ENF (Figure 1) were investigated with the CSAs at different molar ratios. The chiral substrates were used at concentrations of 30 mM and at 10 mM. Given that the AcSiαCD did not show efficiency in enantiodiscrimination, only the data relative to the β- and γ-derivatives are reported and discussed in this work.

#### 2.2.1. Desflurane (DSF)

**Figure 3:**
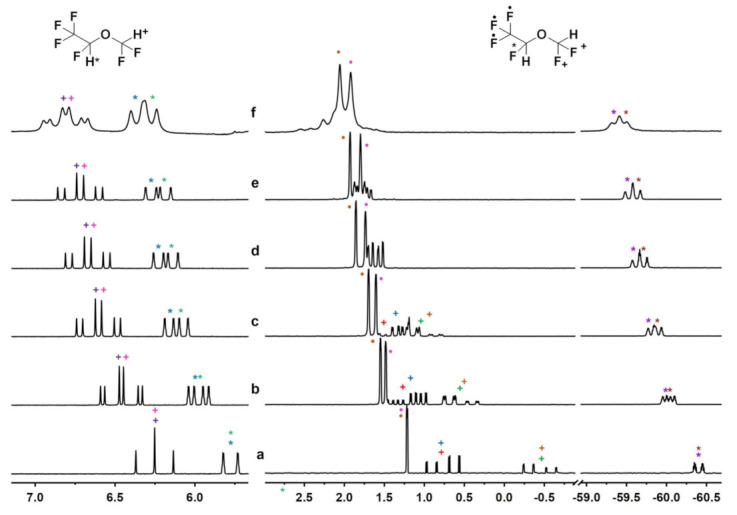
^1^H (600 MHz, 25 °C, C_6_D_12_) (**left**) and ^19^F (564 MHz, 25 °C, C_6_D_12_) (**right**) NMR spectra of DSF (30 mM) alone (**a**) and in the presence of AcSiγCD to give a DSF/CD molar ratio of 8:1 (**b**), 4:1 (**c**), 3:1 (**d**), 2:1 (**e**) and 1:1 (**f**). The different colors help in visualizing the splitting of the signals as a consequence of the interaction with the host.

In the case of DSF, which has a similar chemical structure to ISO (see Figure 1) but with a fluorine atom instead of a chlorine one, the enantiodiscrimination turned out to be more enhanced in the presence of the γ-derivative in this case as well. Similarly to ISO, a baseline separation was already observed at a DSF/AcSiγCD molar ratio of 8-to-1 in the proton belonging to the CHCF_3_ group, and a non-equivalence of 0.036 ppm was detected in the equimolar mixture up to a maximum value of 0.082 ppm (Figure 3 and Table S2 in Supplementary Materials). The proton belonging to the CHF_2_ group underwent considerable enantiodiscrimination as well, starting from non-equivalence values equal to 0.027 ppm for the highest molar ratio (DSF/AcSiγCD 8-to-1) and increasing to 0.043 ppm in the 1-to-1 mixture (Figure 3 and Table S2 in Supplementary Materials). Even higher non-equivalences were observed for the fluorine nuclei, especially the CF_3_ group that gave a non-equivalence of 0.131 ppm in the equimolar mixture (Figure 3 and Table S2 in Supplementary Materials). In the presence of AcSiβCD, lower levels of enantiodiscrimination were detected for both the proton and the fluorine nuclei of the DSF (Table S2 in Supplementary Materials).

The comparison of the non-equivalences measured for the two mixtures ISO/AcSiγCD and DSF/AcSiγCD revealed that the ^1^H and ^19^F nuclei of the CHCF_3_ groups of the two substrates were differentiated to a comparable extent by the macrocycle (Table 1 and Table S2 in Supplementary Materials). At least in the case of ^1^H enantiodiscrimination, the CHF_2_ group of DSF was less efficiently differentiated by the CSA than the ISO; the line broadening observed for the ^19^F resonances, however, did not allow for comparison of the non-equivalence of this group (Table 1 and Table S2 in Supplementary Materials).

#### 2.2.2. Halothane (HAL)

The fluorinated anesthetic HAL, characterized by the presence of a bromine atom instead of a OCHF_2_ group, was enantiodifferentiated by AcSiβCD and AcSiγCD to nearly the same degree but to a lower extent than with ISO and DSF (Table S3 and Figure S2 in Supplementary Materials). Taking into consideration the previously demonstrated [22] relevance of Si-F attractive interactions at the external surface of the cyclodextrins, this kind of behavior can be accounted for by the low number of fluorine atoms in the HAL with respect to the other fluorinated compounds tested in this work.

#### 2.2.3. Enflurane (ENF)

ENF is devoid of the CF_3_ moiety and is the only substrate with a chiral center that shifted to the β-position with respect to the oxygen. Similarly to HAL, a less efficient chiral discrimination was observed for ENF, and no correlation was found between the proton non-equivalences and the CD size. In fact, the differentiations observed for the ^1^H resonances were comparable across the equimolar mixtures of ENF/AcSiβCD and ENF/AcSiγCD (Table S4 and Figure S3 in Supplementary Materials). Concerning the enantiodiscrimination of the fluorine resonances, no differentiation was detected in the presence of the β-derivative. Instead, in the presence of one equivalent of AcSiγCD, non-equivalences of 0.089 ppm/0.071 ppm and 0.030 ppm were measured for the CF_2_ and CHFCl nuclei, respectively (Table S4 in Supplementary Materials). The broadening of the signals of both the substrate and the CSA due to the high CD concentration (30 mM) did not allow for determination of the non-equivalences for the two diastereotopic fluorines belonging to the CHF_2_ group.

#### 2.2.4. Experiments with DSF, HAL, ENF and Compound B in *Sub*-Stoichiometric Conditions

CDs were then tested at *sub*-stoichiometric conditions with DSF, HAL and ENF and at the lower concentration of 10 mM (Tables S2–S4 in Supplementary Materials). As expected, the non-equivalences detected at 10 mM were lower than those obtained at 30 mM, and the enantioseparation decreased by decreasing the content of the CD derivative. For the less enantiodifferentiated compounds, HAL and ENF, *sub*-stoichiometric conditions did not allow sufficient enantiomeric discrimination. Interestingly, in the case of DSF, a baseline separation was observed for the CF_3_ group in the ^19^F NMR spectrum that was already at the DSF/AcSiγCD molar ratio of 4-to-1 (Figure 3 and Table S2 in Supplementary Materials).

Finally, the efficiency of the cyclodextrins in discriminating COMP B in *sub*-stoichiometric conditions was tested at both concentrations (30 mM and 10 mM); the results once more proved the ability of AcSiγCD in the chiral differentiation of the substrate even when used in defect with respect to the chiral compound (Table S5 and Figure S4 in Supplementary Materials). Similarly to the ISO, the non-equivalences measured for the fluorine resonances turned out to be greater than those obtained for the proton ones (Table S5 and Figure S5 in Supplementary Materials). Interestingly, a remarkable baseline separation was achieved between the signals of the two enantiomers already at 8-to-1 substrate-to-AcSiγCD molar ratio. Lowering the concentration did not produce a significant decrease in the values of the non-equivalences, which were still high, so that the quantification of enantiomers was easily able to be performed (Table S5 in Supplementary Materials). It is noteworthy that, as already observed in the proton analysis of an equimolar mixture [22], a significant difference in the linewidth for the signals of the two enantiomers was detected, even in the ^19^F NMR spectrum (Figure S5 in Supplementary Materials).

### 2.3. Interaction Mechanism

**Figure 4:**
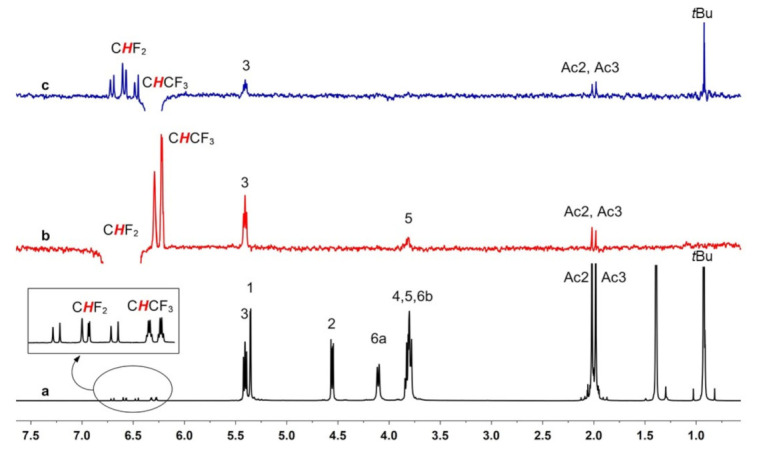
^1^H (600 MHz, 25 °C, C_6_D_12_) NMR spectrum of racemic 1-to-1 ISO/AcSiγCD mixture (**a**, black) and 1D-ROESY (600 MHz, 25 °C, C_6_D_12_, mixing time = 400 ms) spectra of CHF_2_ (**b**, red) and CHCF_3_ (**c**, blue) resonances.

In order to understand the origins of the chiral discrimination phenomena involving each fluorinated substrate and AcSiγCD (the most efficient CSA), intermolecular dipolar interactions were investigated by means of mono-dimensional ROESY experiments. These measurements were performed on equimolar substrate/CD mixtures, by selectively irradiating chosen resonances belonging to the racemic chiral substrate. The analysis of the ISO/AcSiγCD mixture (Figure 4) revealed that the protons of the substrate directly bound to the chlorine atom (CHCF_3_) gave dipolar correlations not only with proton H3 of the cyclodextrin, located on its wider internal part, but also with the derivatizing groups located on the wider rim (OAc) and on the smaller rim (*t*Bu) (Figure 4c). No intermolecular correlations were observed with the H5 proton, located in the smaller part of the cavity. Protons belonging to the CHF_2_ group, instead, produced ROE effects on both H3 and H5 internal protons of AcSiγCD, with a stronger effect on H3 (Figure 4b); no correlation was detected with the silylated portion of the cyclodextrin.

**Figure 5:**
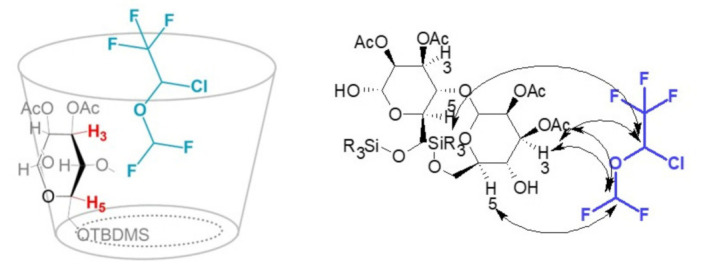
Schematic interaction mechanism between ISO and AcSiγCD (**left**) and representation of the dipolar interactions (indicated by the arrows) detected via ROESY measurements between ISO and the host (**right**).

These results suggested that the interaction mechanism relies on inclusion processes; the guest enters the cavity of the AcSiγCD from the secondary rim, with the CHF_2_ group more deeply included than the CHCF_3_ (Figure 5). The fact that the latter group gave proximity constraints with not only acetyl groups, but also with silyl groups lying on the smaller rim, can be explained based on the previously highlighted cyclodextrin distortion [22] and suggests the occurrence of attractive Si-F intermolecular interactions.

This outcome is in agreement with the non-equivalences measured for ISO in the mixtures with the three macrocycles (Table 1). In the case of AcSiαCD, where no inclusion was possible due to the small cavity sizes, the protons of the substrate could not be discriminated. It is possible to speculate that the absence of inclusion prevented the CF_3_ group from being close to the silyl group of the CD derivative, whereas the CHF_2_ moiety, even if not included, could still be involved in Si-F interactions. The bigger size of the AcSiβCD, even if still not perfectly suitable, allowed the inclusion of ISO, and non-equivalences could be detected for its proton and fluorine nuclei. Finally, the AcSiγCD, which better fit the substrate sizes for an efficient inclusion, guaranteed the best results in terms of enantioseparation.

In the case of the DSF, the inclusion of the substrate into the cavity of the AcSiγCD is proven by the dipolar interactions detected between its methine protons and the inner proton H3 of the CD (Figure S6, Supplementary Materials). However, in this case the inclusion seems to be more superficial than ISO, as no dipolar interactions were detected with proton H5 of the CD. Analogously to ISO, ROE effects were detected between the CHCF_3_ proton and *tert*-butyl moiety of the CD (Figure S6b, Supplementary Materials). Therefore, according to the fact that lower non-equivalences were measured for the CHF_2_ proton of DSF with respect to ISO, a good balance between inclusion and Si-F interactions at the external surface must be reached to have efficient enantiodiscrimination. 

The analysis of the 1D-ROESY spectrum recorded for the ENF/AcSiγCD mixture indicated that the CHF_2_ group of the guest was included in depth, as evidenced by the effect produced both with H3 and H5 nuclei (Figure S7, Supplementary Materials). However, no intermolecular dipolar interaction was found between the ENF protons and silyl moieties lying on the smaller rim of the CD. Thus, in spite of the deep inclusion, the enantiodiscrimination was less efficient than it was for the ISO and DSF due to both the absence of the terminal CF_3_ group and the fact that the chiral center of the guest was in beta position with respect to the included OCHF_2_ moiety.

Finally, only dipolar interactions between the CH protons of the HAL and the acetyl groups were observed, while no ROE effects were detected with H3 nor H5 (Figure S8, Supplementary Materials). The absence of interaction with the inner protons of the host could indicate a superficial interaction mechanism without any inclusion process, and it might explain the scarce non-equivalences measured in the proton and fluorine spectra of the mixtures (Table S4). The presence of a lower number of fluorinated groups on the substrate’s chemical structure, as opposed to the other chiral compounds analyzed, might determine the establishment of weaker Si-F interactions, and, therefore, their scarce enantiodiscriminating efficiency.

## 3. Materials and Methods

### 3.1. Materials

Chiral fluorinated anesthetics isoflurane (ISO), desflurane (DES), halothane (HAL) and enflurane (ENF) were purchased from Merck (Darmstadt, Germany); compound B (COMP B) was kindly provided by Prof. V. Schurig. C_6_D_12_ was purchased from Deutero GmbH (Kastellaun, Germany). All chemicals were used without further purification. The fluorinated anesthetics were handled under a fume hood and under cooling. The sample solutions for the NMR analyses were prepared by mixing the appropriate volume of anesthetic and solvent, and the concentrations were checked via NMR by using an external standard.

### 3.2. ^1^H and ^19^F Characterization of the Fluorinated Substrates

The proton and fluorine NMR spectra of the fluorinated substrates are reported in Supplementary Materials.

#### 3.2.1. Isoflurane

^1^H NMR (600 MHz, C_6_D_12_, 25 °C) δ (ppm): 6.24 (1H, CHF_2_, dd, ^2^J_H-F_ = 72.5 Hz, ^2^J_H-F′_ = 69.8 Hz), 5.90 (1H, CHCF_3_, qd, ^3^J_H-F_ = 4.2 Hz, ^4^J_H-F′_ = 0.9 Hz). 

^19^F NMR (564 MHz, C_6_D_12_, 25 °C) δ (ppm): 5.11 (3F, CF_3_, dd, ^3^J_F-H_ = 4.2 Hz, ^5^J_F-F′_ = 2.0 Hz), −1.40 (1F, F of CHF_2_, dd, ^2^J_F-F′_ = 160.6 Hz, ^2^J_F-H_ = 72.5 Hz), −2.33 (1F, F′ of CHF_2_, ddqd, ^2^J_F′-F_ = 160.6 Hz, ^2^J_F′-H_ = 69.8 Hz, ^5^J_F′-F_ = 2.0 Hz, ^4^J_F′-H_ = 0.9 Hz).

#### 3.2.2. Desflurane

^1^H NMR (600 MHz, C_6_D_12_, 25 °C) δ (ppm): 6.25 (1H, CHF_2_, t, ^2^J_H-F_ = 70.6 Hz), 5.78 (1H, CHFCF_3_, dq, ^2^J_H-F_ = 54.3 Hz, ^3^J_H-F_ = 3.1 Hz).

^19^F NMR (564 MHz, C_6_D_12_, 25 °C) δ (ppm): 5.11 (3F, CF_3_, ddd, 3J_F-F_ = 6.0 Hz, ^3^J_F-H_ = 3.1 Hz, ^5^J_F-F_ = 1.2 Hz), 0.84 (1F, F of CHF_2_, ddd, ^2^J_F-F′_ = 160.2 Hz, ^2^J_F-H_ = 70.6 Hz, ^4^J_F-F_ = 8.0 Hz), −0.45 (1F, F′ of CHF_2_, ddd, ^2^J_F′-F_ = 160.2 Hz, ^2^J_F′-H_ = 70.6 Hz, ^4^J_F′-F_ = 5.0 Hz), −60.4 (1F, CHF, ddqd, ^2^J_F-H_ = 54.3 Hz, ^4^J_F-F_ = 8.0 Hz, ^3^J_F-F_ = 6.0 Hz, ^4^J_F-F′_ = 5.0 Hz).

#### 3.2.3. Halothane

^1^H NMR (600 MHz, C_6_D_12_, 25 °C) δ (ppm): 5.62 (1H, CH, q, ^3^J_H-F_ = 5.2 Hz).

^19^F NMR (564 MHz, C_6_D_12_, 25 °C) δ (ppm): 9.28 (3F, CF_3_, d ^3^J_F-H_ = 5.2 Hz).

#### 3.2.4. Enflurane

^1^H NMR (600 MHz, C_6_D_12_, 25 °C) δ (ppm): 6.56 (1H, CHF_2_, t, ^2^J_H-F_ = 70.1 Hz), 5.96 (1H, CHFCl, dt, ^2^J_H-F_ = 48.2 Hz, ^3^J_H-F_ = 4.3 Hz).

^19^F NMR (564 MHz, C_6_D_12_, 25 °C) δ (ppm): 1.09 (2F, CHF_2_, dt, ^2^J_F-H_ = 70.1 Hz, ^4^J_F-F_ = 4.4 Hz), 0.31 (1F, F of CF_2_, ddq, ^2^J_F-F′_ = 143.7 Hz, ^3^J_F-F_ = 11.7 Hz, ^4^J_F-F_ = 4.4 Hz), −0.02 (1F, F′ of CF_2_, ddq, ^2^J_F′-F_ = 143.7 Hz, ^3^J_F′-F_ = 11.7 Hz, ^4^J_F′-F_ = 4.4 Hz), −69.03 (1F, CHFCl, dt, ^2^J_F-H_ = 48.2 Hz, ^3^J_F-F_ = ^3^J_F-F′_ = 11.7 Hz).

#### 3.2.5. Compound B

^19^F NMR (564 MHz, C_6_D_12_, 25 °C) δ (ppm): 11.89 (3F, CF3, m), 2.95 (1F, F of CF_2_, m), 0.80 (1F, F′ of CF_2_, m), −69.26 (1F, F of CH_2_F, m).

### 3.3. Synthesis of Silylated-Acetylated Cyclodextrins

Hexakis(2,3-di-*O*-acetyl-6-*O*-*tert*-butyldimethylsilyl)-α-cyclodextrin (AcSiαCD), heptakis(2,3-di-*O*-acetyl-6-*O*-*tert*-butyldimethylsilyl)-β-cyclodextrin (AcSiβCD) and octakis(2,3-di-*O*-acetyl-6-*O*-*tert*-butyldimethylsilyl)-γ-cyclodextrin (AcSiγCD) were synthesized according to the synthetic steps shown in Scheme S1 in the Supplementary Materials. Native cyclodextrins were first regioselectively silylated on the primary side and then exhaustively acetylated on the secondary side. The synthesis of the per(6-*O*-tert-butyldimethylsilyl)-α-, β- and γ-cyclodextrin derivatives was achieved according to a previously developed procedure [28]. 

Per(2,3-di-*O*-acetyl-6-*O*-tert-butyldimethylsilyl)-cyclodextrins. A suspension of per(6-*O*-tert-butyldimethylsilyl)-cyclodextrins (α: 14.1 g-8.5 mmol; β: 16.4 g-8.5 mmol; γ: 18.8 g-8.5 mmol) and a catalytic amount of 4-DMAP (~5 mg) in a mixture of Ac_2_O (100 mL) and dry pyridine (200 mL) was stirred under N_2_ at room temperature for 24 h, at which point a clear solution was formed and the TLC (EtOAc) showed the presence of one product (Rf = 0.42 (α), 0.45 (β), 0.48 (γ)). The solution was poured into a mixture of 5% aqueous HCl (3 L) and ice, stirred until the ice melted and extracted with CH_2_Cl_2_ (2 × 1 L). The combined organic phases were washed with H_2_O (2 × 500 mL), dried (MgSO_4_) and concentrated. Traces of pyridine were removed by co-evaporation with toluene (2 × 500 mL). The resulting material was mixed with silica gel (30 g), suspended in CH_2_Cl_2_, and the solvent was removed under vacuum. Chromatography (20:1 EtOAc–hexane) was used to produce a material that was further purified by precipitation from 1:10 Me_2_CO–H_2_O to yield the title compounds (α: 15.8 g, 7.31 mmol, 86%; β: 17.36 g, 6.88 mmol, 81%; γ: 22.5 g, 7.82 mmol, 92%) as white amorphous solids. For elemental analysis, the material was reprecipitated from freshly distilled Me_2_CO (2 mL) and hexane (10 mL), and dried at 100 °C under high vacuum for 3 h; α: [α]_D_^25^ +109 (c 1.0, CH_2_Cl_2_), lit. +109 (c 1.0, CHCl_3_); β: [α]_D_^25^ +107 (c 1.0, CH_2_Cl_2_), lit. +107 (c 1.0, CHCl_3_); [α]_D_^25^ +108 (c 1.0, CH_2_Cl_2_), lit. +1097 (c 1.0, CHCl_3_); Rf (EtOAc) = 0.42 (α), 0.45 (β), 0.48 (γ); IR (ATR): 2960, 1738, 1369, 1213, 1027, 734 cm^−1^. ^1^H and ^13^C NMR characterization data have been reported in reference [22].

### 3.4. Methods

NMR measurements were carried out in C_6_D_12_ on a Varian (Palo Alto, CA, USA) INOVA600 spectrometer equipped with a 5 mm reverse probe operating at 600 MHz and 564 MHz for ^1^H and ^19^F nuclei, respectively; the temperature was controlled (25 ± 0.1 °C). The proton and fluorine chemical shifts were referred to tetramethylsilane (TMS) and trifluorotoluene, respectively, as the external standards. The proton 1D-ROESY spectra were recorded using a selective inversion pulse, 1024 transients, 32K points, a relaxation delay of 3 s, a gain of 20 and a mixing time of 400 ms; the π pulse was optimized for each sample that was analyzed. The pulse sequence 1D ROSEY (dpfgse), available in the VARIAN library, was chosen for the experiments.

## 4. Conclusions

Two main contributions can be envisaged in the present work, the first one dealing with the development of an analytical method based on the use of lipophilic silylated-acetylated cyclodextrins for the NMR differentiation of fluorinated chiral anesthetics. The second contribution goes into the heart of enantiodifferentiation driven by cyclodextrin hosts. In fact, it was clearly highlighted that a deep inclusion in the CD cavity is not a fundamental prerequisite in NMR enantiodiscrimination processes involving cyclodextrin derivatives such as CSAs. The effect of the derivatizing groups, so far scarcely considered, may, indeed, reflect the conformational features of the CDs, thus bringing about distortions from the truncated-cone-shaped structure, which could favor the occurrence of attractive interactions between included guests and derivatizing groups lying on the two rims of the cyclodextrins, even in the cases of compounds that are not deeply included.

Cyclodextrin derivatives endowed with acetyl and silyl groups proved to be able to originate the NMR discrimination of fluorinated chiral substrates by exploiting synergic and well-balanced contributions from both partial inclusions in the CD cavity and attractive intermolecular Si-F interactions occurring at the external surface of the host. The latter were favored by the relevant degree of rotation toward the glycosidic linkages of the CD in deuterated cyclohexane. 

AcSiγCD was found to be the best chiral agent among the three analogue derivatives, probably because it provided the highest number of silyl moieties for Si-F interactions. The contribution of the cavity size to enantiodiscrimination processes was witnessed due to the fact that the smaller cyclodextrin (AcSiαCD), which was not suited to host the fluorinated substrates, only produced a negligible amount of proton enantiomeric differentiation. The AcSiβCD displayed less efficient enantiodiscrimination because it contained a lower number of silylated glucopyranose rings than the AcSiγCD. Additionally, the relevance of the number of derivatized glucopyranose rings is supported by the fact that efficient NMR enantiodifferentiation was also detected in *sub*-stoichiometric amounts of the host.

## Figures and Tables

**Table 1:** ^1^H (600 MHz, C_6_D_12_, 25 °C) and ^19^F (564 MHz, C_6_D_12_, 25 °C) NMR non-equivalences (|δ*_R_* − δ*_S_*|, ppm) measured for ISO (30 mM) resonances in the presence of AcSiαCD, AcSiβCD or AcSiγCD at different molar ratios.

Concentration (mM)	Molar Ratio ISO/AcSiCD	|δ*_R_* − δ*_S_*|			
		^1^H		^19^F	
AcSiαCD		**CHCF_3_**	C**H**F_2_	C**F_3_**	CH**F_2_**
5	6:1	-	-	0.006	0.010/0.006
10	3:1	-	-	0.007	0.022/0.010
15	2:1	-	-	0.007	0.032/0.015
30	1:1	-	-	nd *	0.054/0.025
AcSiβCD					
4.3	7:1	0.006	0.006	0.020	0.019/0.008
8.6	3.5:1	0.007	0.010	0.033	0.039/0.013
15	2:1	0.007	0.013	0.044	0.059/0.016
30	1:1	0.013	0.019	0.071	0.121/0.018
AcSiγCD					
3.75	8:1	0.049	0.045	0.075	0.653/0.535
7.5	4:1	0.071	0.065	0.116	1.12/0.894
11.25	3:1	0.081	0.074	0.137	1.45/1.15
15	2:1	0.081	0.075	0.143	1.66/1.31
30	1:1	0.083	0.082	0.165	2.08/1.98

* not determined.

## Data Availability

Data is contained within the article or Supplementary Materials.

## Supplementary Materials

The following supporting information can be downloaded at https://www.mdpi.com/article/10.3390/molecules28062804/s1: Scheme S1: Synthetic strategy for silylated-acetylated cyclodextrins; Figure S1: 1H (600 MHz, 30 mM, 25 °C, C6D12) NMR spectra of racemic ISO (a) alone and (b) in the presence of AcSiγCD to give a ISO/CD molar ratio of 16:1; Table S1: 1H (600 MHz, C6D12, 25 °C) and 19F (564 MHz, C6D12, 25 °C) NMR non-equivalences (|δR − δS|, ppm) measured for ISO resonances in the presence of equimolar amounts of AcSiαCD, AcSiβCD or AcSiγCD at 10 mM and 30 mM concentration; Table S2: 1H (600 MHz, C6D12, 25 °C) and 19F (564 MHz, C6D12, 25 °C) NMR non-equivalences (|δR − δS|, ppm) of DSF (30 mM) in the presence of AcSiβCD or AcSiγCD at different molar ratios; the results reported in parenthesis refer to the 10 mM concentration; Table S3: 1H (600 MHz, C6D12, 25 °C) and 19F (564 MHz, C6D12, 25 °C) NMR non-equivalences (|δR − δS|, ppm) of HAL (30 mM) in the presence of AcSiγCD at different molar ratios; the results reported in parenthesis refer to the 10 mM concentration; Figure S2: 1H (600 MHz, 25 °C, C6D12) and 19F (564 MHz, 25 °C, C6D12) NMR spectra of HAL (30 mM) alone (a, f), in the presence of (left) AcSiβCD to give a HAL/CD molar ratio of 7:1 (b), 3.5:1 (c), 2:1 (d) and 1:1 (e), and in the presence of (right) AcSiγCD to give a HAL/CD molar ratio of 8:1 (g), 4:1 (h), 3:1 (i), 2:1 (j) and 1:1 (k); Table S4: 1H (600 MHz, C6D12, 25 °C) and 19F (564 MHz, C6D12, 25 °C) NMR non-equivalences (|δR − δS|, ppm) of ENF (30 mM) in the presence of AcSiβCD or AcSiγCD at different molar ratio; the results reported in parenthesis refer to the 10 mM concentration; Figure S3: 1H (600 MHz, 25 °C, C6D12) NMR spectra of ENF (30 mM) alone (a, f), in the presence of (left) AcSiβCD to give a ENF/CD molar ratio of 7:1 (b), 3.5:1 (c), 2:1 (d) and 1:1 (e), and in the presence of (right) AcSiγCD to give a ENF/CD molar ratio of 8:1 (g), 4:1 (h), 3:1 (i), 2:1 (j) and 1:1 (k); Table S5: 1H (600 MHz, C6D12, 25 °C) and 19F (564 MHz, C6D12, 25 °C) NMR non-equivalences (|δR − δS|, ppm) of COMP B (30 mM) in the presence of AcSiβCD or AcSiγCD at different molar ratios; the results reported in parenthesis refer to the 10 mM concentration; Figure S4: 1H (600 MHz, 25 °C, C6D12) NMR spectra of COMP B (30 mM) alone (a) and in the presence of AcSiγCD to give a COMP B/CD molar ratio of 8:1 (b), 4:1 (c), 3:1 (d) and 2:1 (e). The other resonances belong to CSA; Figure S5: 19F (564 MHz, 25 °C, C6D12) NMR spectra of COMP B (30 mM) alone (a) and in the presence of AcSiγCD to give a COMP B/CD molar ratio of 8:1 (b), 4:1 (c), 3:1 (d) and 2:1 (e); Figure S6: 1H (600 MHz, 25 °C, C6D12) NMR spectrum of racemic 1:1 DSF/AcSiγCD mixture (a) and 1D-ROESY (600 MHz, 25 °C, C6D12, mixing time = 400 ms) spectra of CHCF3 (b) and CHF2 (c) resonances; Figure S7: 1H (600 MHz, 25 °C, C6D12) NMR spectrum of racemic 1:1 ENF/AcSiγCD mixture (a) and 1D-ROESY (600 MHz, 25 °C, C6D12, mixing time = 400 ms) spectra of CHF2 (b) and CHFCl (c) resonances; Figure S8: 1H (600 MHz, 25 °C, C6D12) NMR spectrum of racemic 1:1 HAL/AcSiγCD mixture (a) and 1D-ROESY (600 MHz, 25 °C, C6D12, mixing time = 400 ms) spectrum of CHClBr (b) resonance; 1H and 19F NMR characterization data of ISO, DSF, HAL and ENF.

## Sample Availability

Not available.
