# Supplementary material for: Silylated-Acetylated Cyclodextrins as Chiral Sensors for the Enantiodiscrimination of Fluorinated Anesthetics
Source: Molecules. 2023 Mar 20;28(6):2804. doi: 10.3390/molecules28062804 (PMC10058779; doi:10.3390/molecules28062804)
Supplement: Supplementary file 1 [file molecules-28-02804-s001.zip › molecules-2286388-supplementary.pdf]

Silylated-acetylated cyclodextrins as chiral sensors for the enantiodiscrimination of fluorinated anesthetics

Alessandra Recchimurzo [1], Federica Balzano [1,*], Gloria Uccello Barretta [1,*], Luca Gherardi [1], Milo Malanga [2] and Federica Aiello [3]

[1] Department of Chemistry and Industrial Chemistry, University of Pisa, Via G. Moruzzi 13, 56124 Pisa, Italy; alessandra.recchimurzo@phd.unipi.it (A.R.); luca.gherardi@phd.unipi.it (L.G.)
[2] CycloLab, Cyclodextrin R&D Ltd, Budapest, H-1097 Illatos út 7, Hungary; milomalanga@gmail.com (M.M.)
[3] National Research Council, Institute for Chemical and Physical Processes (CNR-IPCF), Via G. Moruzzi 1, 56124 Pisa, Italy; federica.aiello@cnr.it
* Correspondence: federica.balzano@unipi.it (F.B.); gloria.uccello.barretta@unipi.it (G.U.B.); Tel.: +390502219232 (F.B., G.U.B.)

Supplementary Material

Table of contents

Scheme S1. Synthetic strategy for silylated-acetylated cyclodextrins.

Figure S1. ^{1}H (600 MHz, 30 mM, 25 °C, C_6D_{12}) NMR spectra of racemic ISO (a) alone and (b) in the presence of AcSiγCD to give a ISO/CD molar ratio of 16:1.

Table S1. ^{1}H (600 MHz, C_6D_{12}, 25 °C) and ^{19}F (564 MHz, C_6D_{12}, 25 °C) NMR non-equivalences ($|\delta_R - \delta_S|$, ppm) measured for ISO resonances in the presence of equimolar amounts of AcSiαCD, AcSiβCD or AcSiγCD at 10 mM and 30 mM concentration.

Table S2. ^{1}H (600 MHz, C_6D_{12}, 25 °C) and ^{19}F (564 MHz, C_6D_{12}, 25 °C) NMR non-equivalences ($|\delta_R - \delta_S|$, ppm) of DSF (30 mM) in the presence of AcSiβCD or AcSiγCD at different molar ratios; the results reported in parenthesis refer to the 10 mM concentration.

Table S3. ^{1}H (600 MHz, C_6D_{12}, 25 °C) and ^{19}F (564 MHz, C_6D_{12}, 25 °C) NMR non-equivalences ($|\delta_R - \delta_S|$, ppm) of HAL (30 mM) in the presence of AcSiγCD at different molar ratios; the results reported in parenthesis refer to the 10 mM concentration.

Figure S2. ^{1}H (600 MHz, 25 °C, C_6D_{12}) and ^{19}F (564 MHz, 25 °C, C_6D_{12}) NMR spectra of HAL (30 mM) alone (a, f), in the presence of (left) AcSiβCD to give a HAL/CD molar ratio of 7:1 (b), 3.5:1 (c), 2:1 (d) and 1:1 (e), and in the presence of (right) AcSiγCD to give a HAL/CD molar ratio of 8:1 (g), 4:1 (h), 3:1 (i), 2:1 (j) and 1:1 (k).

Table S4. ^{1}H (600 MHz, C_6D_{12}, 25 °C) and ^{19}F (564 MHz, C_6D_{12}, 25 °C) NMR non-equivalences ($|\delta_R - \delta_S|$, ppm) of ENF (30 mM) in the presence of AcSiβCD or AcSiγCD at different molar ratio; the results reported in parenthesis refer to the 10 mM concentration.

Figure S3. ^{1}H (600 MHz, 25 °C, C_6D_{12}) NMR spectra of ENF (30 mM) alone (a, f), in the presence of (left) AcSiβCD to give a ENF/CD molar ratio of 7:1 (b), 3.5:1 (c), 2:1 (d) and 1:1 (e), and in the presence of (right) AcSiγCD to give a ENF/CD molar ratio of 8:1 (g), 4:1 (h), 3:1 (i), 2:1 (j) and 1:1 (k).

Table S5. ^1H (600 MHz, C_6D_{12}, 25 °C) and ^{19}F (564 MHz, C_6D_{12}, 25 °C) NMR non-equivalences ($|\delta_R - \delta_S|$, ppm) of COMP B (30 mM) in the presence of AcSiβCD or AcSiγCD at different molar ratios; the results reported in parenthesis refer to the 10 mM concentration.

Figure S4. ^1H (600 MHz, 25 °C, C_6D_{12}) NMR spectra of COMP B (30 mM) alone (a) and in the presence of AcSiγCD to give a COMP B/CD molar ratio of 8:1 (b), 4:1 (c), 3:1 (d) and 2:1 (e). The other resonances belong to CSA.

Figure S5. ^{19}F (564 MHz, 25 °C, C_6D_{12}) NMR spectra of COMP B (30 mM) alone (a) and in the presence of AcSiγCD to give a COMP B/CD molar ratio of 8:1 (b), 4:1 (c), 3:1 (d) and 2:1 (e).

Figure S6. ^1H (600 MHz, 25 °C, C_6D_{12}) NMR spectrum of racemic 1-to-1 DSF/AcSiγCD mixture (a) and 1D-ROESY (600 MHz, 25 °C, C_6D_{12}, mixing time = 400 ms) spectra of $CHCF_3$ (b) and CHF_2 (c) resonances.

Figure S7. ^1H (600 MHz, 25 °C, C_6D_{12}) NMR spectrum of racemic 1-to-1 ENF/AcSiγCD mixture (a) and 1D-ROESY (600 MHz, 25 °C, C_6D_{12}, mixing time = 400 ms) spectra of CHF_2 (b) and CHFCl (c) resonances.

Figure S8. ^1H (600 MHz, 25 °C, C_6D_{12}) NMR spectrum of racemic 1-to-1 HAL/AcSiγCD mixture (a) and 1D-ROESY (600 MHz, 25 °C, C_6D_{12}, mixing time = 400 ms) spectrum of CHClBr (b) resonance.

^1H and ^{19}F NMR spectra of ISO, DSF, HAL and ENF and ^{19}F NMR spectrum of COMP B; the ^1H NMR spectrum of COMP B is reported in reference [22].

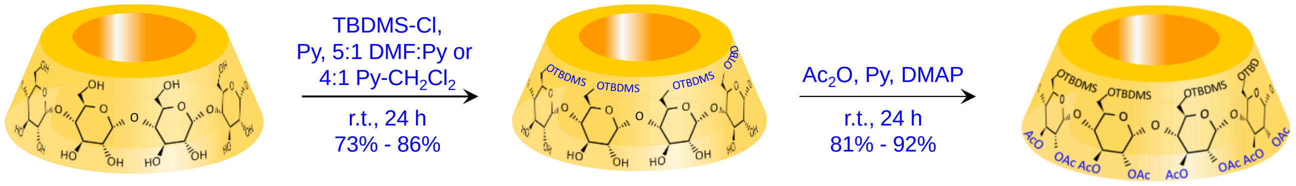

Scheme S1: Synthetic strategy for silylated-acetylated cyclodextrins.

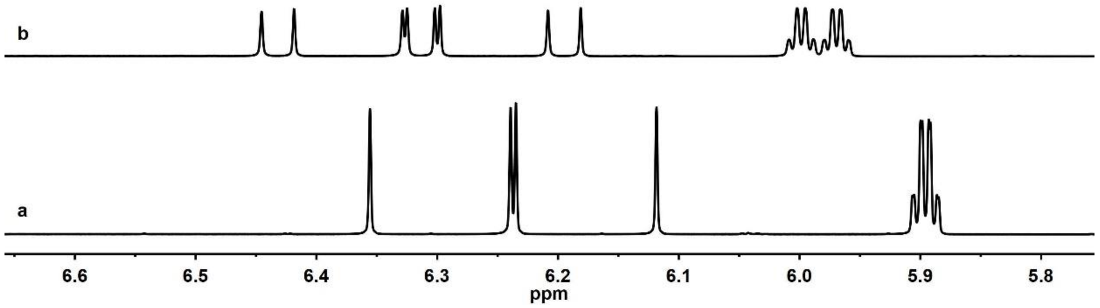

Figure S1. ^{1}H (600 MHz, 30 mM, 25 °C, C_6D_{12}) NMR spectra of racemic ISO (a) alone and (b) in the presence of AcSiγCD to give a ISO/CD molar ratio of 16:1.

Table S1. ^{1}H (600 MHz, C_6D_{12}, 25 °C) and ^{19}F (564 MHz, C_6D_{12}, 25 °C) NMR non-equivalences ($|\delta_R - \delta_S|$, ppm) measured for ISO resonances in the presence of equimolar amounts of AcSiαCD, AcSiβCD or AcSiγCD at 10 mM and 30 mM concentration.

		$|\delta_R - \delta_S|$			
		^{1}H		^{19}F	
	**mM**	$CHCF_3$	CHF_2	CF_3	CHF_2
**AcSiαCD**	30	-	-	nd*	0.054/0.025
	10	-	-	0.003	0.024/0.012
**AcSiβCD**	30	0.013	0.019	0.071	0.121/0.018
	10	0.006	0010	0.038	0.056/0.013
**AcSiγCD**	30	0.083	0.082	0.165	2.08/1.98
	10	0.068	0.055	0.127	1.53/1.11

* not determined

Table S2. ^{1}H (600 MHz, C_6D_{12}, 25 °C) and ^{19}F (564 MHz, C_6D_{12}, 25 °C) NMR non-equivalences ($|\delta_R - \delta_S|$, ppm) of DSF (30 mM) in the presence of AcSiβCD or AcSiγCD at different molar ratios; the results reported in parenthesis refer to the 10 mM concentration.

**Molar ratio sub:CSA**	$|\delta_R - \delta_S|$				
	^{1}H		^{19}F		
	$CHCF_3$	CHF_2	CF_3	CHF_2	CHF

AcSiβCD	7:1	0.009 (-)	0.004 (-)	0.024 (0.016)	0.027/0.023 (0.012/0.014)	- (-)
	3.5:1	0.011 (-)	0.007 (-)	0.033 (0.018)	0.041/0.042 (0.015/0.018)	- (-)
	2:1	0.014 (-)	0.008 (-)	0.048 (0.021)	0.056/0.063 (0.026/0.025)	- (-)
	1:1	0.021 (0.014)	0.010 (0.008)	0.065 (0.050)	0.070/0.099 (0.044/0.040)	- (-)
AcSiγCD	8:1	0.036 (0.013)	0.027 (0.008)	0.063 (0.020)	0.062/0.024 (0.024/nd*)	0.048 (0.012)
	4:1	0.058 (0.024)	0.038 (0.017)	0.092 (0.060)	0.077/0.030 (0.052/0.040)	0.067 (0.041)
	3:1	0.062 (0.035)	0.043 (0.025)	0.119 (0.062)	-/- (0.053/0.043)	0.088 (0.043)
	2:1	0.065 (0.043)	0.043 (0.031)	0.128 (0.080)	nd/nd (0.061/0.054)	0.095 (0.055)
	1:1	0.082 (0.052)	0.043 (0.038)	0.131 (nd)	nd/nd (nd/0.064)	0.096 (nd)

* not determined.

Table S3. ^{1}H (600 MHz, C_6D_{12}, 25 °C) and ^{19}F (564 MHz, C_6D_{12}, 25 °C) NMR non-equivalences ($|\delta_R - \delta_S|$, ppm) of HAL (30 mM) in the presence of AcSiγCD at different molar ratios; the results reported in parenthesis refer to the 10 mM concentration.

	Molar ratio sub:CSA	$|\delta_R - \delta_S|$ CH	$|\delta_R - \delta_S|$ CF_3
AcSiβCD	7:1	0.002 (0.001)	0.006 (0.001)
	3.5:1	0.003 (0.001)	0.009 (0.004)
	2:1	0.005 (0.001)	0.012 (0.005)
	1:1	0.008 (0.003)	0.023 (0.009)
AcSiγCD	8:1	0.002 (0.001)	0.002 (-)
	4:1	0.005 (0.002)	0.005 (-)
	3:1	0.007 (0.003)	0.007 (0.003)
	2:1	0.009 (0.004)	0.009 (0.004)
	1:1	0.014 (0.009)	0.013 (0.008)

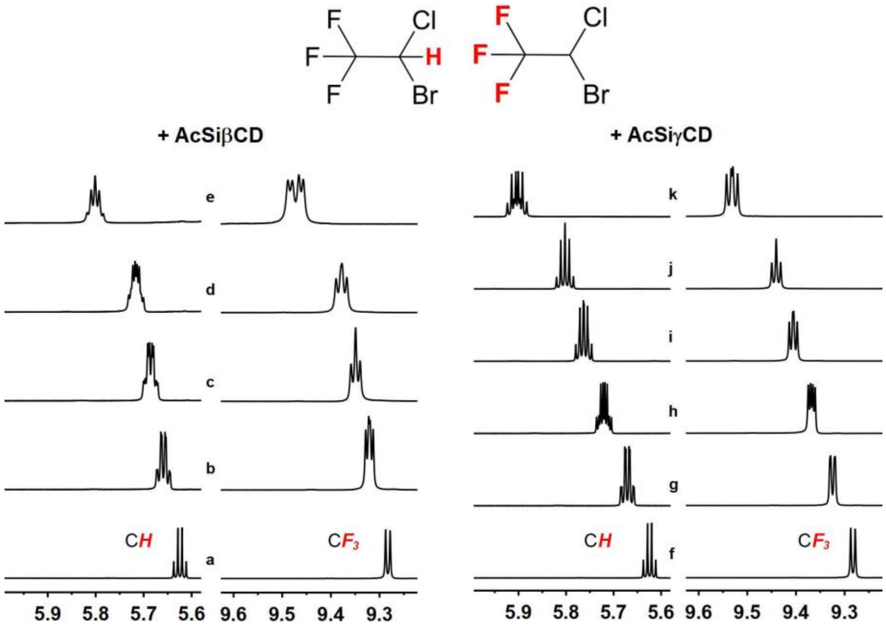

Figure S2. ^{1}H (600 MHz, 25 °C, C_6D_{12}) and ^{19}F (564 MHz, 25 °C, C_6D_{12}) NMR spectra of HAL (30 mM) alone (a, f), in the presence of (left) AcSiβCD to give a HAL/CD molar ratio of 7:1 (b), 3.5:1 (c), 2:1 (d) and 1:1 (e), and in the presence of (right) AcSiγCD to give a HAL/CD molar ratio of 8:1 (g), 4:1 (h), 3:1 (i), 2:1 (j) and 1:1 (k).

Table S4. ^{1}H (600 MHz, C_6D_{12}, 25 °C) and ^{19}F (564 MHz, C_6D_{12}, 25 °C) NMR non-equivalences ($|\delta_R - \delta_S|$, ppm) of ENF (30 mM) in the presence of AcSiβCD or AcSiγCD at different molar ratio; the results reported in parenthesis refer to the 10 mM concentration.

	Molar ratio sub:CSA	$|\delta_R - \delta_S|$				
		^{1}H		^{19}F		
		CHCl	CHF_2	CF_2	CHFCl	CHF_2
**AcSiβCD**	7:1	0.002	0.002	-	-	nd*
		(-)	(-)	(-)	(-)	(nd)
	3.5:1	0.003	0.005	-	-	nd
		(-)	(-)	(-)	(-)	(nd)
	2:1	0.004	0.004	-	-	nd
		(-)	(-)	(-)	(-)	(nd)
	1:1	0.007	0.005	-	-	nd
		(0.002)	(0.003)	(-)	(-)	(nd)
**AcSiγCD**	8:1	0.002	-	-/-	0.010	nd
		(-)	(-)		(-)	(nd)
	4:1	0.004	-	nd/nd	0.020	nd
		(-)	(-)	(-/-)	(0.006)	(nd)
	3:1	0.006	-	0.025/0.021	0.020	nd
		(0.002)	(-)	(-/-)	(0.009)	(nd)
	2:1	0.007	-	0.041/0.038	0.021	nd
		(0.004)	(-)	(-/-)	(0.017)	(nd)
	1:1	0.007	0.004	0.089/0.071	0.030	nd
		(0.006)	(-)	(0.027/0.026)	(0.019)	(nd)

* not determined.

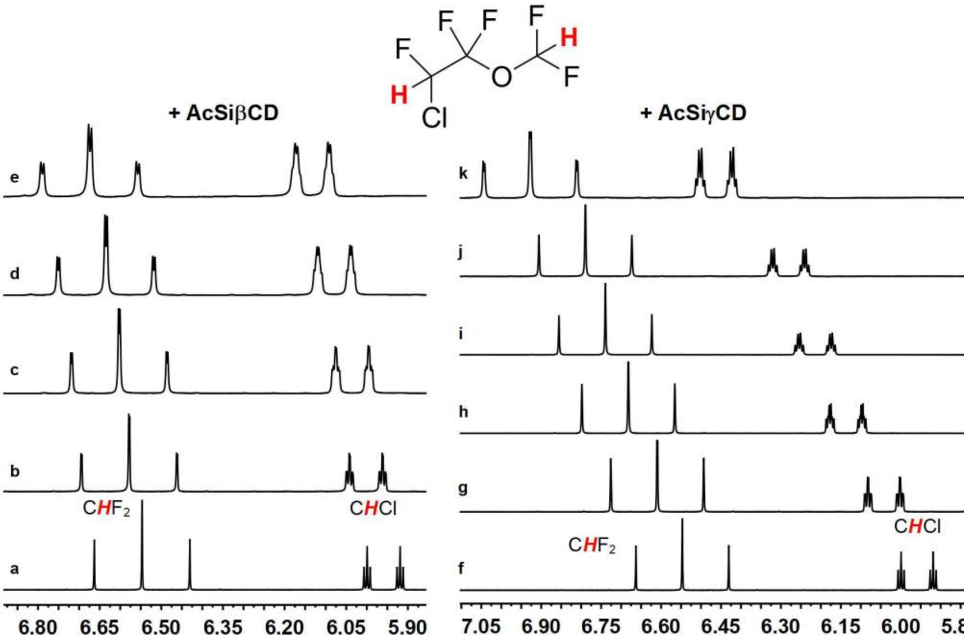

Figure S3. ^{1}H (600 MHz, 25 °C, C_6D_{12}) NMR spectra of ENF (30 mM) alone (a, f), in the presence of (left) AcSiβCD to give a ENF/CD molar ratio of 7:1 (b), 3.5:1 (c), 2:1 (d) and 1:1 (e), and in the presence of (right) AcSiγCD to give a ENF/CD molar ratio of 8:1 (g), 4:1 (h), 3:1 (i), 2:1 (j) and 1:1 (k).

Table S5. ^{1}H (600 MHz, C_6D_{12}, 25 °C) and ^{19}F (564 MHz, C_6D_{12}, 25 °C) NMR non-equivalences ($|\delta_R - \delta_S|$, ppm) of COMP B (30 mM) in the presence of AcSiβCD or AcSiγCD at different molar ratios; the results reported in parenthesis refer to the 10 mM concentration.

	**Molar ratio sub:CSA**	**$|\delta_R - \delta_S|$**					
		**^{1}H**			**^{19}F**		
		CH_2	CH	OCH_3	CF_2	CH_2**F**	CF_3
**AcSiβCD**	7:1	0.007/0.007	0.009	0.002	0.038/0.066	0.036	0.016
		(0.004/0.004)	(0.004)	(-)	(0.006/0.005)	(0.023)	(0.006)
	3.5:1	0.013/0.015	0.020	0.004	nd*/0.128	0.073	0.038
		(0.005/0.005)	(0.010)	(0.001)	(0.063/0.047)	(0.038)	(0.015)
	2:1	nd/nd	0.029	0.006	0.116/0.190	0.098	0.056
		(nd/nd)	(0.014)	(0.003)	(0.103/0.067)	(0.062)	(0.019)
**AcSiγCD**	8:1	0.075/0.065	0.103	0.041	1.021/0.93	0.778	0.169
		(0.065/0.058)	(0.116)	(0.037)	(0.82/0.67)	(0.672)	(0.138)
	4:1	nd/nd	0.182	0.074	1.85/1.56	1.41	0.303
		(nd/0.102)	(0.187)	(0.062)	(1.39/1.18)	(1.08)	(0.223)
	3:1	0.177/0.174	0.336	0.104	2.47/2.03	1.81	0.380
		(0.144/0.143)	(0.293)	(0.092)	(2.09/1.74)	(1.63)	(0.326)
	2:1	0.210/0.207	0.394	0.115	3.02/2.39	2.10	0.423
		(0.182/nd)	(0.344)	(0.106)	(2.55/2.14)	(1.91)	(0.388)

* not determined

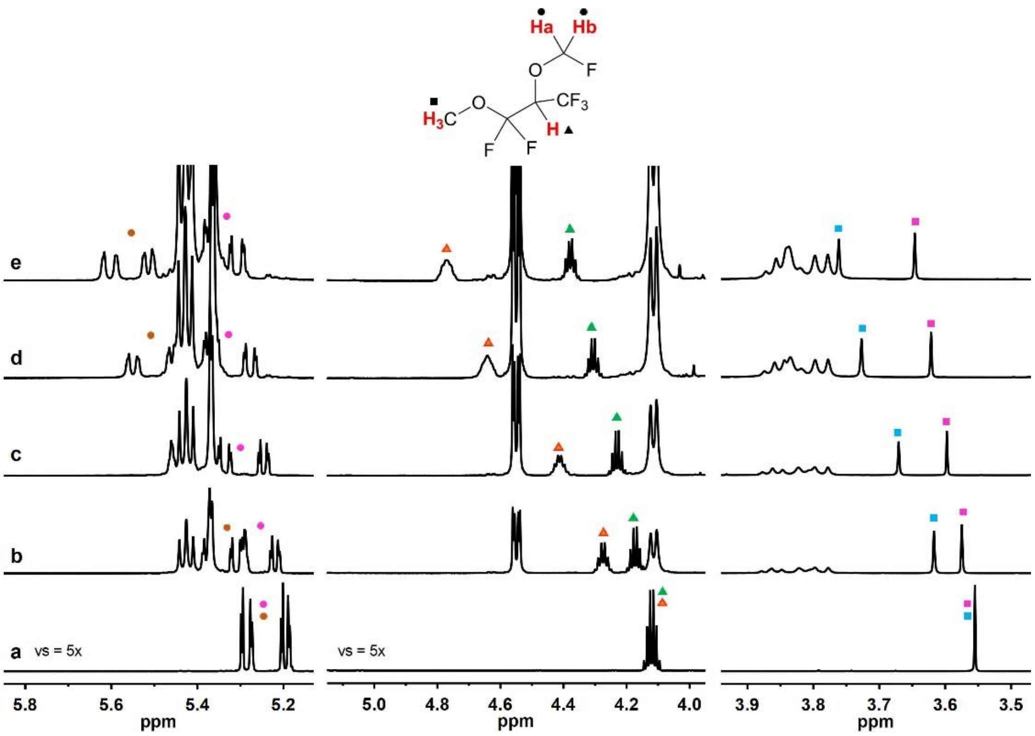

Figure S4. ^{1}H (600 MHz, 25 °C, C_6D_{12}) NMR spectra of COMP B (30 mM) alone (a) and in the presence of AcSiγCD to give a COMP B/CD molar ratio of 8:1 (b), 4:1 (c), 3:1 (d) and 2:1 (e). The other resonances belong to CSA.

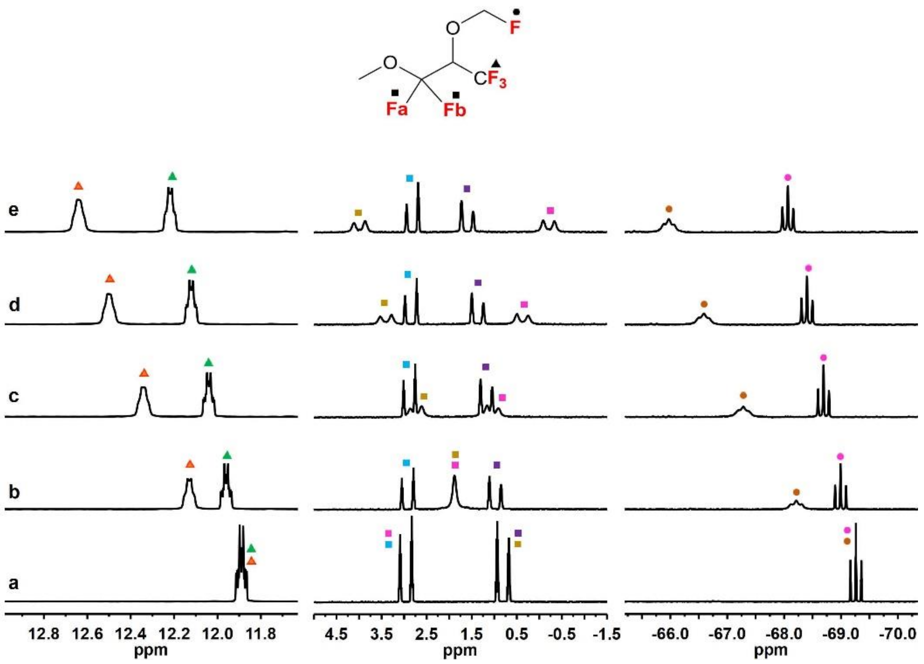

Figure S5. ^{19}F (564 MHz, 25 °C, C_6D_{12}) NMR spectra of COMP B (30 mM) alone (a) and in the presence of AcSiγCD to give a COMP B/CD molar ratio of 8:1 (b), 4:1 (c), 3:1 (d) and 2:1 (e).

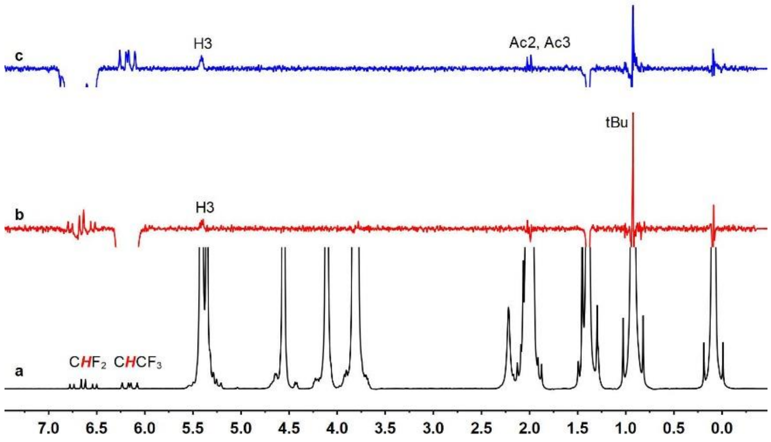

Figure S6. ^{1}H (600 MHz, 25 °C, C_6D_{12}) NMR spectrum of racemic 1-to-1 DSF/AcSiγCD mixture (a) and 1D-ROESY (600 MHz, 25 °C, C_6D_{12}, mixing time = 400 ms) spectra of $CHCF_3$ (b) and CHF_2 (c) resonances.

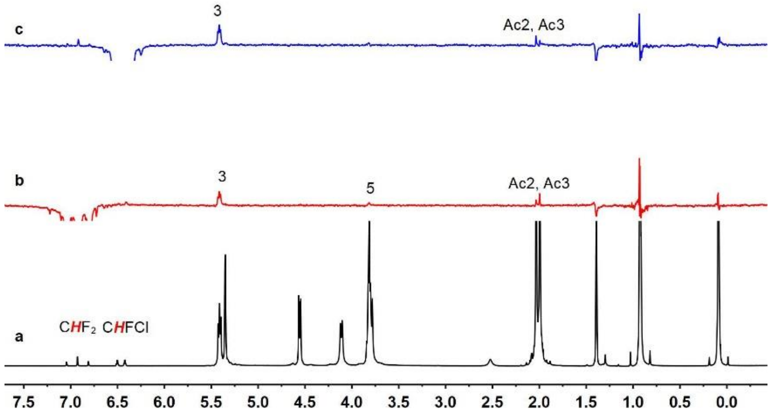

Figure S7. ^{1}H (600 MHz, 25 °C, C_6D_{12}) NMR spectrum of racemic 1-to-1 ENF/AcSiγCD mixture (a) and 1D-ROESY (600 MHz, 25 °C, C_6D_{12}, mixing time = 400 ms) spectra of CHF_2 (b) and CHFCl (c) resonances.

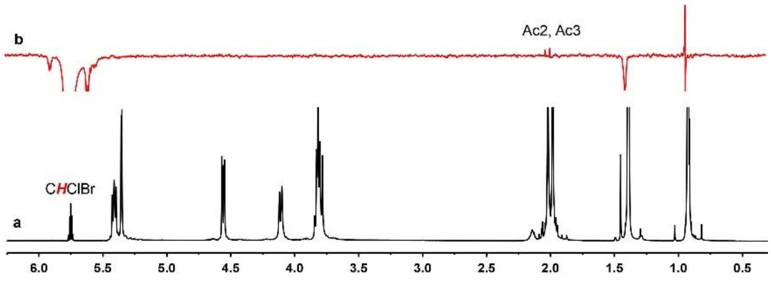

Figure S8. ^{1}H (600 MHz, 25 °C, C_6D_{12}) NMR spectrum of racemic 1-to-1 HAL/AcSiγCD mixture (a) and 1D-ROESY (600 MHz, 25 °C, C_6D_{12}, mixing time = 400 ms) spectrum of CHClBr (b) resonance.

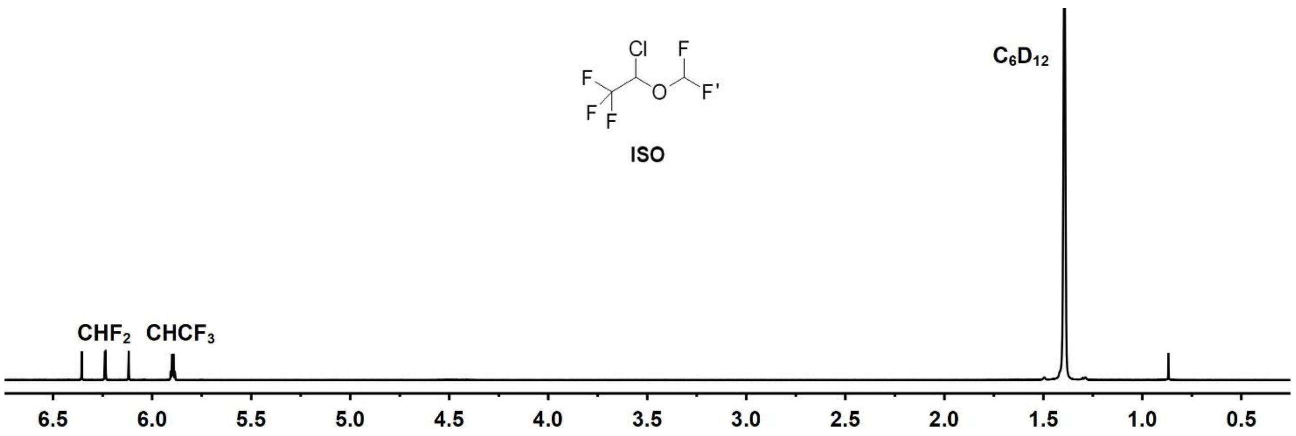

^{1}H NMR (600 MHz, C_6D_{12}, 25 °C) spectrum of ISO.

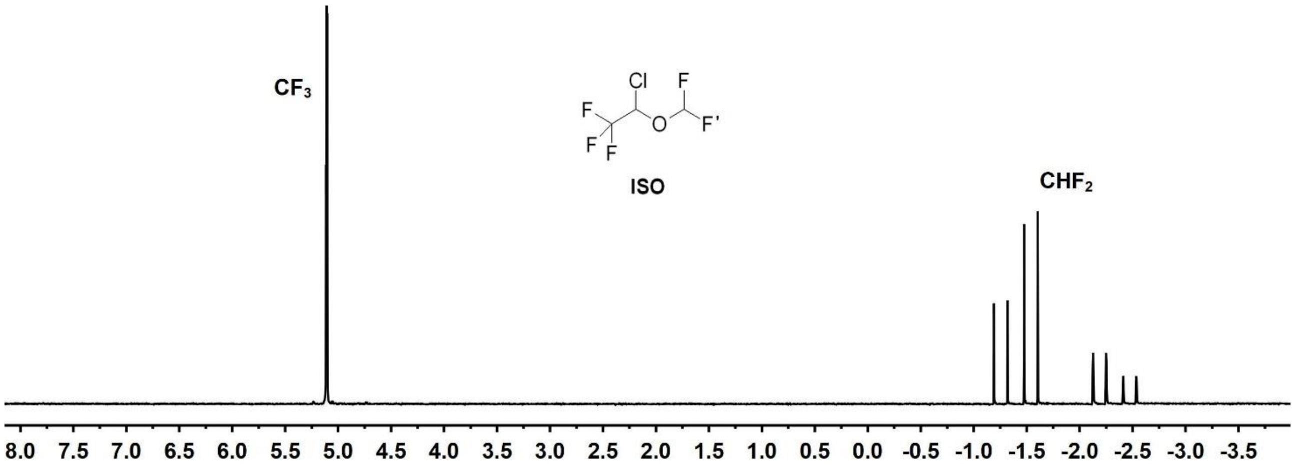

^{19}F NMR (564 MHz, C_6D_{12}, 25 °C) spectrum of ISO.

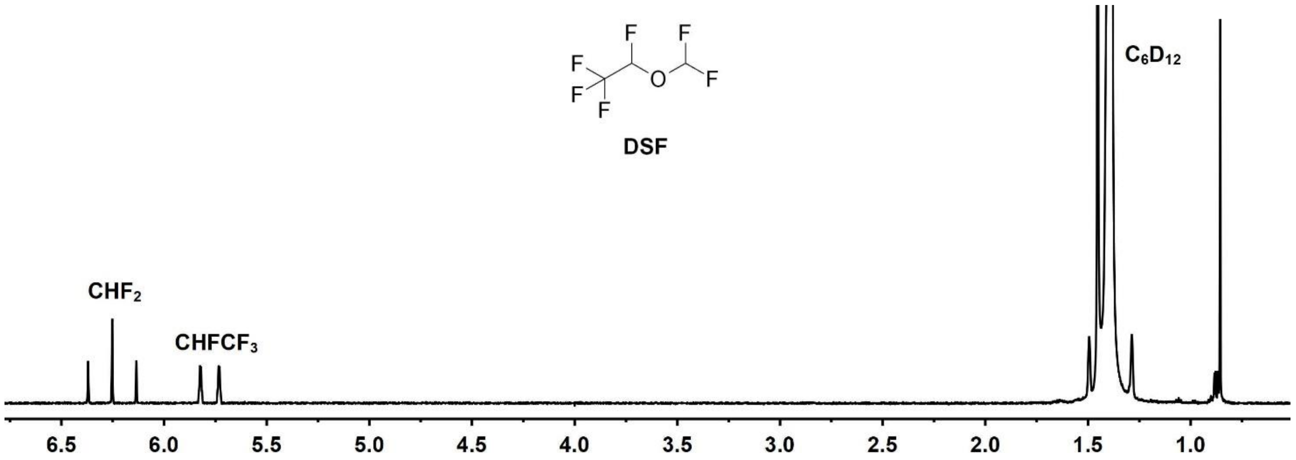

^{1}H NMR (600 MHz, C_6D_{12}, 25 °C) spectrum of DSF.

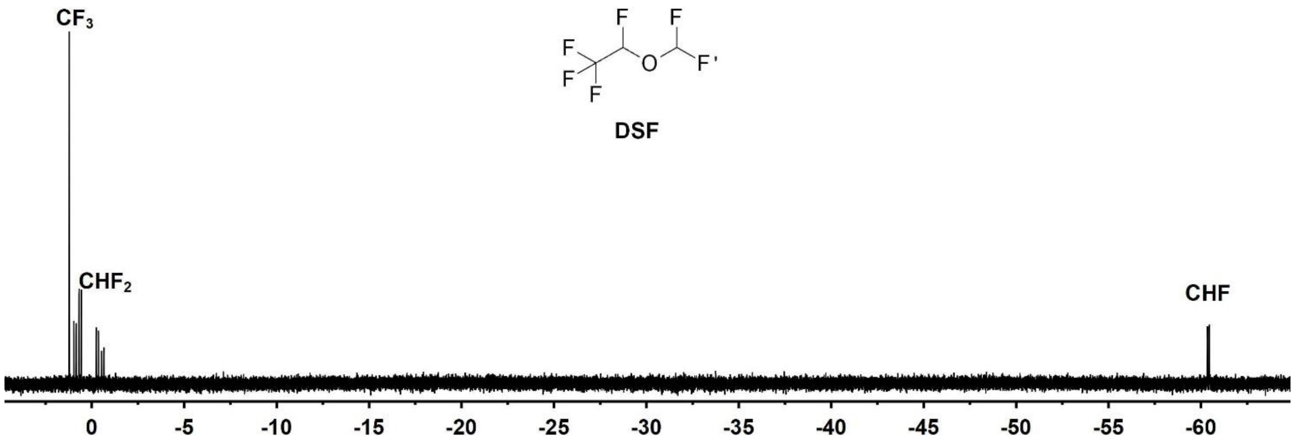

^{19}F NMR (564 MHz, C_6D_{12}, 25 °C) spectrum of DSF.

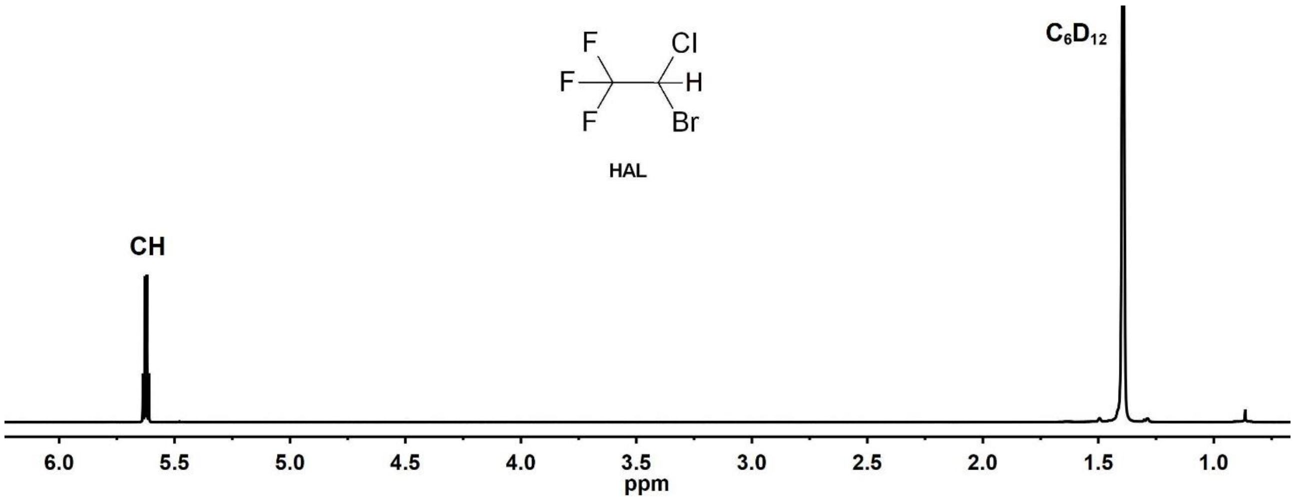

^{1}H NMR (600 MHz, C_6D_{12}, 25 °C) spectrum of HAL.

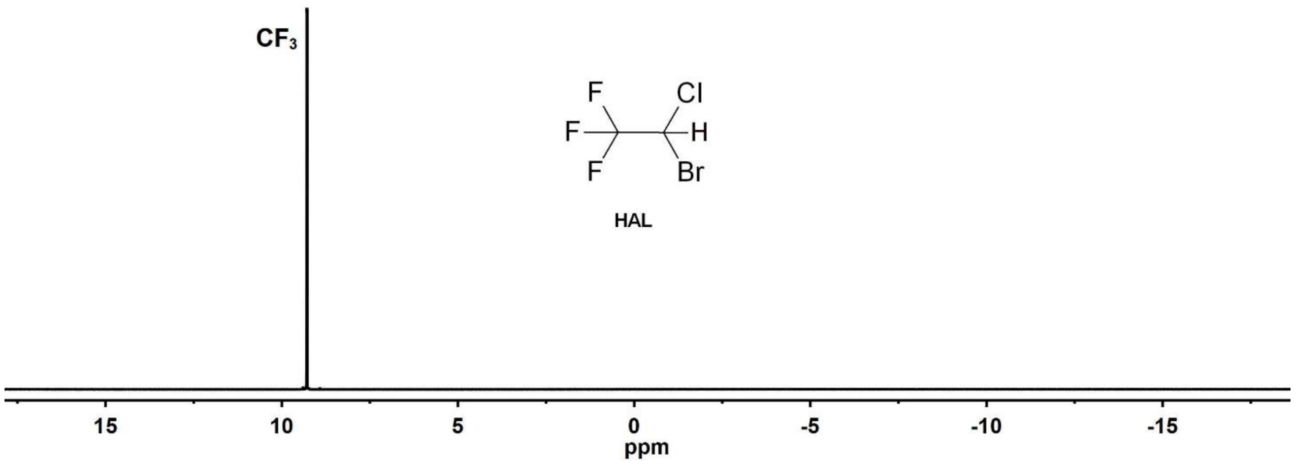

^{19}F NMR (564 MHz, C_6D_{12}, 25 °C) spectrum of HAL.

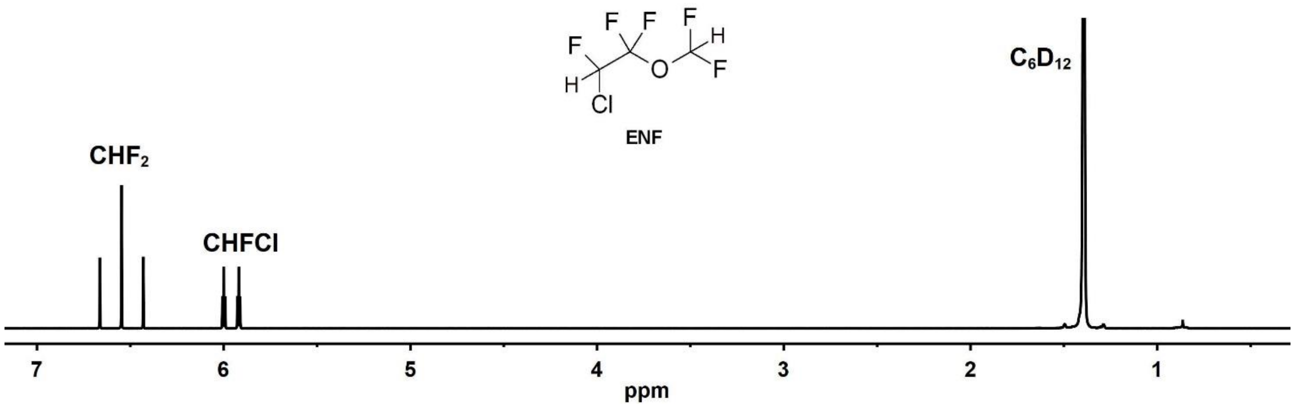

^1H NMR (600 MHz, C_6D_{12}, 25 °C) spectrum of ENF.

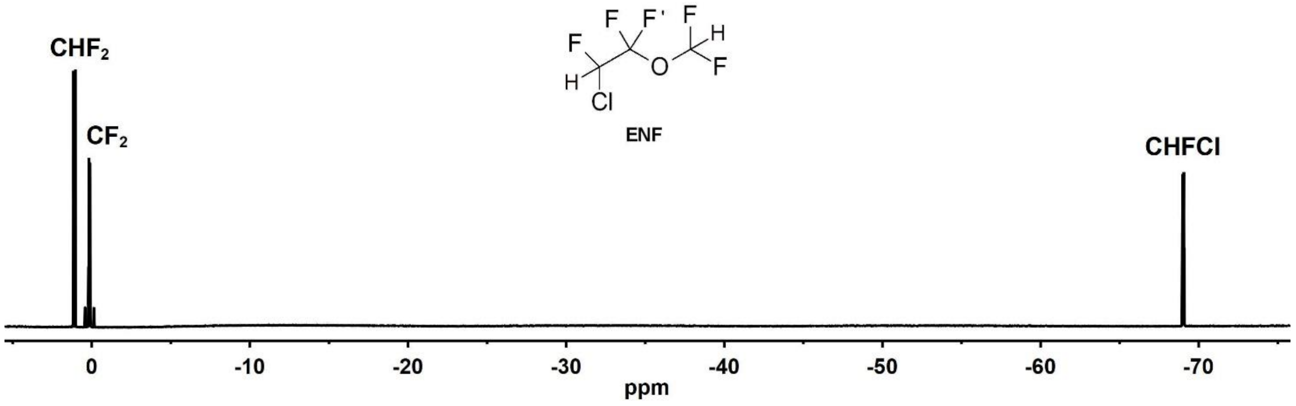

^{19}F NMR (564 MHz, C_6D_{12}, 25 °C) spectrum of ENF.

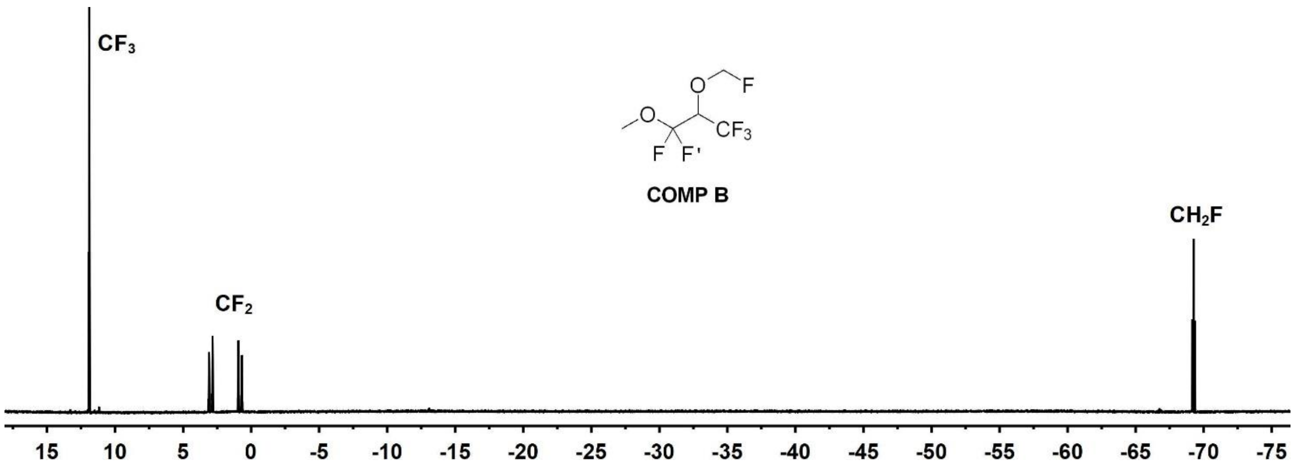

^{19}F NMR (564 MHz, C_6D_{12}, 25 °C) spectrum of COMP B.
